# Development of a robust protocol for gene expression analysis using formalin-fixed, paraffin-embedded liver transplant biopsy specimens

**DOI:** 10.1136/jclinpath-2013-201548

**Published:** 2013-06-11

**Authors:** Emily Thompson, Alastair D Burt, Catriona E Barker, John A Kirby, John G Brain

**Affiliations:** Applied Immunobiology and Transplantation Research Group, Faculty of Medical Sciences, Institute of Cellular Medicine, Medical School, Newcastle University, Newcastle upon Tyne, Tyne and Wear, UK

**Keywords:** Transplantation, Liver, Biliary

## Abstract

Liver transplant biopsies are routinely archived following formalin fixation and paraffin embedding and may provide an additional source of diagnostic information following transcriptomic biomarker analysis. This study was designed to compare gene transcription between resting and stressed biliary cells in culture, these cells after fixation and embedding and archival liver transplant biopsy tissue. The transcription of p21/WAF1 and transforming growth factor (TGF)-β1 showed similar changes in the fresh and embedded liver cells. However, the expression of TGF-β2 was markedly different between the fresh and embedded samples, suggesting that fixation can produce sequence-specific artefacts. Sufficient quantities of pure RNA were recovered from all the liver transplant biopsies to allow complementary DNA production. Measurement of the transcription of all three genes showed variability between the cases. Although the results for individual transcripts should be interpreted with care, these data do suggest the feasibility of performing a larger biomarker discovery studies using archival tissue.

## Introduction

Liver transplant biopsy sections are routinely assessed with standard histological or immunohistochemical staining.^1^ However, there is a clear potential to recover improved diagnostic and prognostic information from allograft tissues by quantitative analysis of gene expression,^2^ which is unrealised. In order to minimise invasive liver biopsy procedures with their associated mortality and morbidity, it would be advantageous if the RNA for such research and analyses could be reliably isolated from the formalin-fixed, paraffin-embedded (FFPE) biopsy tissues used for histology.

Formalin fixation can modify nucleic acids by the addition of monomethylol groups to the bases.^3^ While this limits the recovery of full-length messenger RNA (mRNA), TaqMan chemistry allows gene transcription to be quantified by real-time PCR analysis of short complementary DNA (cDNA) sequences derived from fragmented mRNA.^4^ Another potential problem includes the unknown effects of oxidative degradation during block storage. This can be somewhat minimised by RNA isolation soon after fixation and by removing the most damaged superficial tissue.^5^

Despite these methodological advances, there has been no validation of systematic studies for the potential for gene expression analysis of FFPE human liver specimens. The current study was designed to examine the expression of a range of transplantation-relevant genes in resting and stressed biliary epithelial cells (BECs) before and after formalin fixation and paraffin embedding and to assess the applicability of these techniques for FFPE liver transplant biopsy samples.

## Materials and methods

### Cells and stimulation

The immortalised BEC line H69^6^ was cultured to confluence. Resting cells were compared against stressed cultures that had 200 µM H_2_O_2_ added to the medium for 2 h; the cells were subsequently washed and returned to normal medium for 4, 8, 12, 24 and 36 h. RNA was isolated from resting and stressed cells using TRI reagent (Sigma).

### Cell fixation and paraffin embedding

Three cell pellets were created: resting cells, 12-hours post-H_2_O_2_ injury and 24 hours post-H_2_O_2_ injury. Cells were suspended in 10 mL of 10% neutral buffered formalin overnight at 4°C. The cells were then centrifuged and the pellets embedded in paraffin.

### Tissue samples

Six times zero (post-reperfusion) FFPE liver transplant biopsy specimens were used in this study. These tissue samples were from separate patients transplanted before 2006 in accordance with Research Ethics Committee approval (06/Q0905/150).

### FFPE mRNA isolation and validation

RNA was isolated from FFPE liver tissue using an RNeasy FFPE kit (Qiagen). For each reaction, 4×10 µm sections were freshly cut from the FFPE blocks; the outer 40 µm was discarded to minimise the effects of oxidation. The purity and quantity of each RNA sample was determined by Nanodrop spectrophotometry; RNA integrity was validated by agarose electrophoresis (50 mL TAE buffer, 0.5 g agarose and 3 µL Ethidium Bromide) and an AlphaImager (Alpha Innotech) to identify the 18 S and 28 S subunits of ribosomal RNA.

### cDNA synthesis and TaqMan real-time PCR

The RNA was reverse transcribed to cDNA using an AffinityScript Multi-Temperature cDNA synthesis kit (Agilent Technologies). TaqMan chemistry was used with exon-spanning primers (Applied Biosystems) and a StepOne Plus real-time PCR system (Applied Biosystems) to quantify gene expression. The cycle threshold (*C*_t_) for genes of interest (p21/WAF1, transforming growth factor (TGF)-β1 and TGF-β2) was normalised using the housekeeping gene glyceraldehyde-3-phosphate dehydrogenase to allow calculation of fold changes in gene expression.^7^

## Results

**Figure 1:**
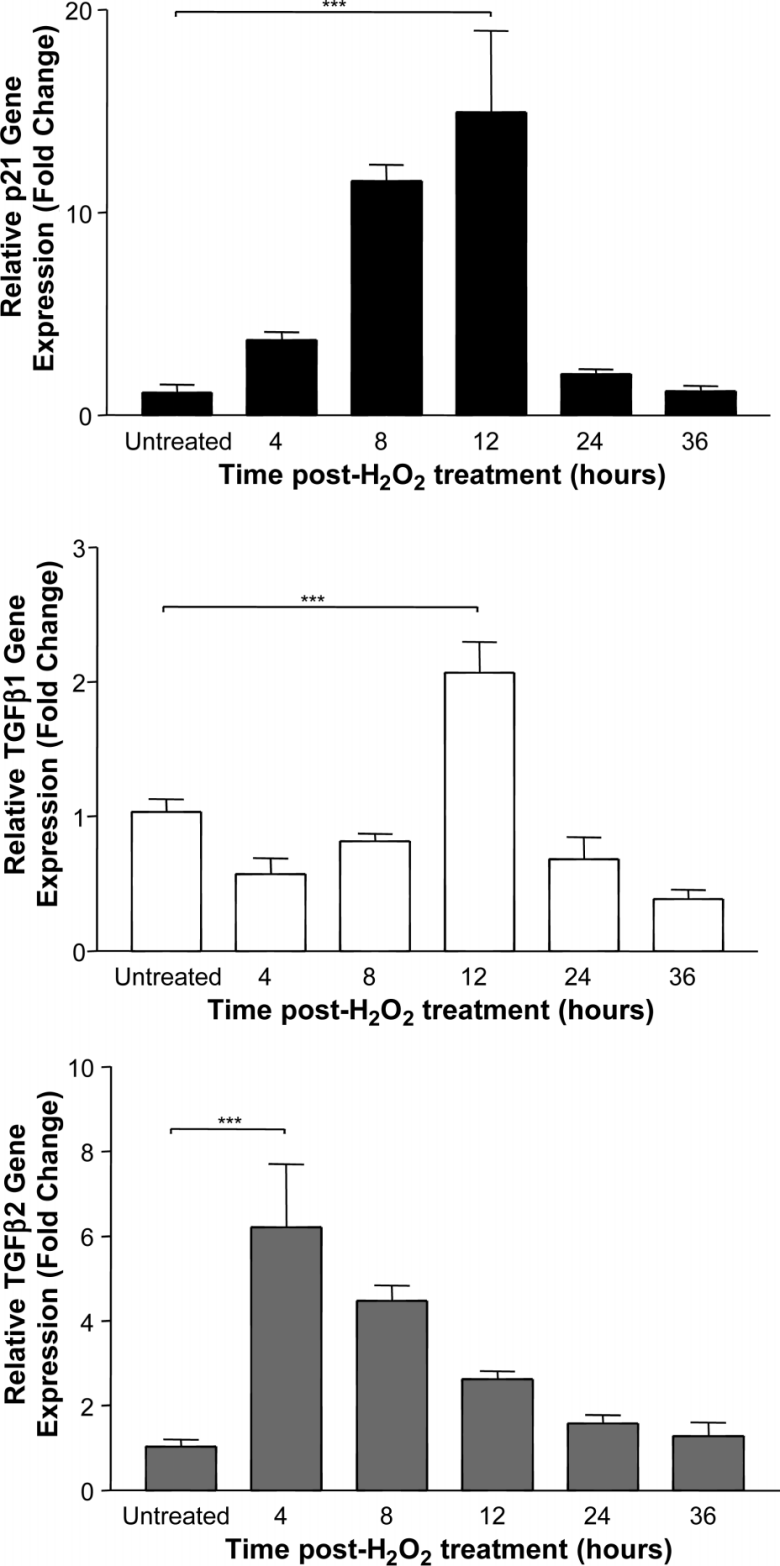
Measurement of gene expression by cultured human biliary epithelial cells at various times after 2 h of oxidative stress (***p<0.001). Summary data of three separate experiments are shown.

**Figure 2:**
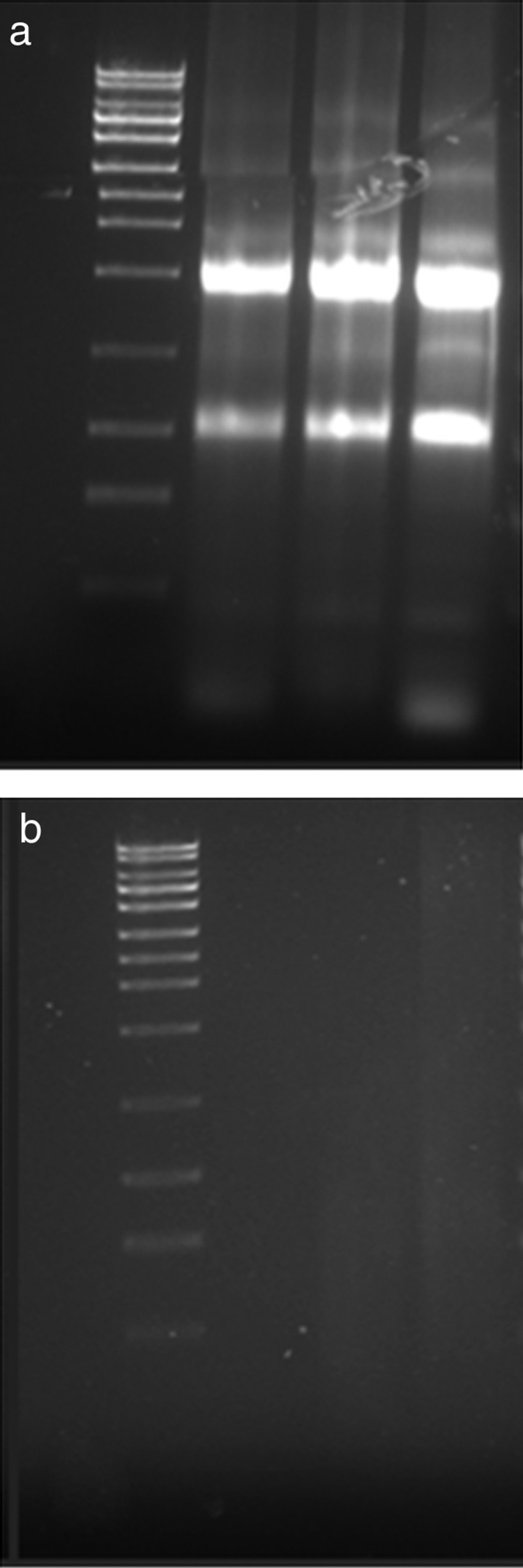
Representative gel showing RNA extracted from non-fixed (A) and formalin-fixed, paraffin-embedded  processing (B) biliary epithelial cells; non-degraded 18S and 28S ribosomal RNA bands can be seen in panel (A).

**Figure 3:**
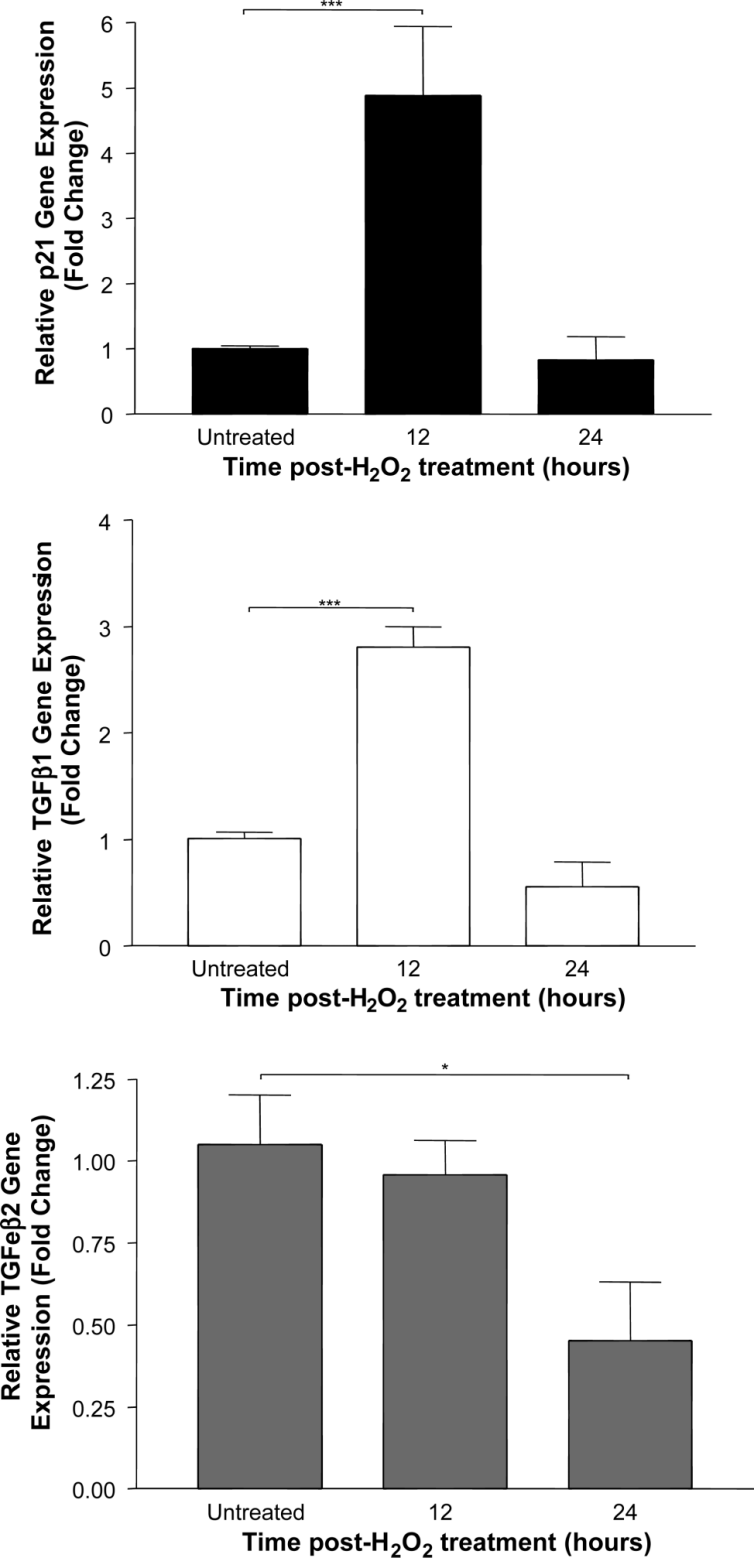
Measurement of gene expression by cultured human biliary epithelial cells at various times after a brief period of oxidative stress and following formalin-fixed, paraffin-embedded processing (***p<0.001). Summary data of three separate experiments are shown.

Experiments in vitro showed that oxidative stress induced a significant increase in the expression of p21/WAF1, TGF-β1 and TGF-β2 by BEC (figure 1; all p<0.001), with maximal upregulation observed after 12, 12 and 4 h, respectively. The RNA extracted from these cells before and after oxidative stress for 4 and 12 h (figure 2A) showed intact 18S and 28S ribosomal RNA subunits with no evidence of degradation. However, although the RNA-extracted FFPE cell pellets at these time points had a reasonable purity (typical A260/280=2.03 indicating little protein contamination, and A260/230=2.02 indicating little solvent contamination), none of these samples showed discrete 18S or 28S bands (figure 2B). Gene expression analysis of these samples showed a significant increase in both p21/WAF1 and TGF-β1 12 h after oxidative stress (figure 3; both p<0.001). However, no expected increase was observed in expression of the TGF-β2 transcript.

**Figure 4:**
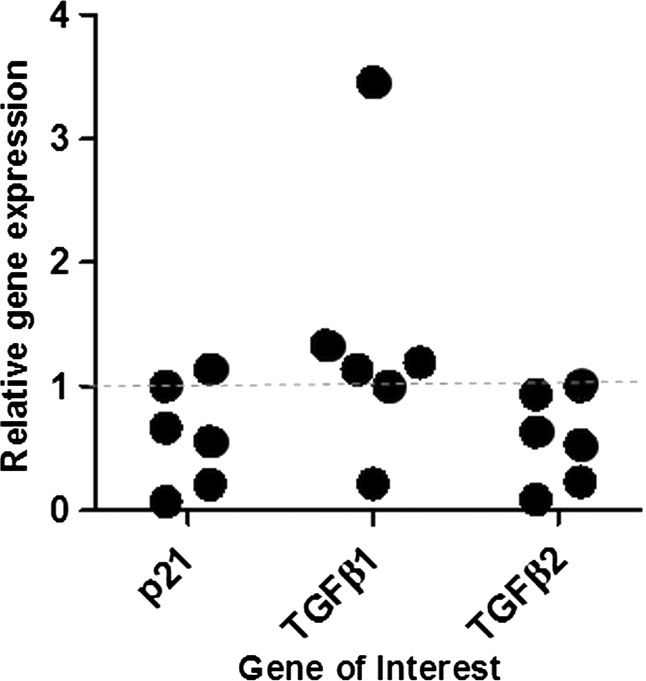
Gene expression analysis using formalin-fixed, paraffin-embedded biopsy-derived RNA from six liver transplant cases; all biopsy samples were taken at time zero immediately after reperfusion. All results were normalised to one arbitrarily chosen sample.

A quantity (2.95±0.42 µg; mean±SEM) of pure RNA (260/280 and 260/230 ratios were all above 2) sufficient for cDNA production was isolated from sections of each of the six FFPE liver biopsy samples. After reverse transcription, each sample showed exponential amplification during real-time PCR generating measurable *C*_t_ values. One of the six cases was arbitrarily chosen to normalise the summary data presented in figure 4.

## Discussion

This study was designed to assess the feasibility of performing gene transcription analysis using archival FFPE liver biopsy material. Initial experiments defined the increase in expression of mRNA encoding the senescence marker p21/WAF1 and the fibrogenic–immunoregulatory growth factors TGF-β1 and TGF-β2 by cultured human BEC following a brief period of oxidative stress. Results from this study were compared with similarly stressed cells after formalin fixation and embedding in paraffin.

Pure RNA could be extracted from the cultured cells after FFPE processing. However, the integrity of this RNA was certainly damaged and neither 18S nor 28S ribosomal RNA sequences could be identified. Real-time PCR analysis of cDNA generated from this RNA demonstrated a similar stress-induced increase in p21/WAF1 and TGF-β1 to that observed in fresh cells. Importantly, however, gene expression results for TGF-β2 showed markedly different results between the fresh and FFPE cell pellets, with the latter cells indicating a decrease in expression of this gene. This result can be interpreted as providing evidence for sequence-specific damage produced by the FFPE process, suggesting that careful validation should always be performed before extending this analysis to RNA from biopsy material.

It was possible reliably to isolate sufficient quantities of pure RNA from sections cut from all six archival FFPE liver transplant biopsy blocks used in this study. After cDNA synthesis, the housekeeping gene and each of the three genes of interest could be detected during real-time PCR amplification. This study suggests the feasibility of performing a larger study incorporating biopsy material taken at later time points following liver transplantation. There have been previous studies looking into mRNA extraction from tumour tissue banks^8^ and in some types of liver disease with no attempt to investigate the effect of the FFPE process upon mRNA expression. The data from this study would indicate that such a study in liver samples should only be attempted after careful validation of individual mRNA stability by in vitro modelling.

**Key messages**

- mRNA of sufficient quality for analysis by real-time PCR can be recovered reproducibly from FFPE human liver tissue.
- Sequence specific mRNA degradation can occur during FFPE processing.
- There is evidence of heterogeneity in specific gene expression between different FFPE transplant liver specimens.
